# No Sex Differences in Psychological Burden and Health Behaviors of Healthcare Workers During the COVID-19 Stay-at-Home Orders

**DOI:** 10.3389/fmed.2021.740064

**Published:** 2021-10-12

**Authors:** Wenli Gu, Xiao Liu, Runlu Sun, Yuan Jiang, Zhengyu Cao, Maoxiong Wu, Jianyong Ma, Zhiteng Chen, Yangxin Chen, Yuling Zhang, Jingfeng Wang

**Affiliations:** ^1^Department of Cardiology, Sun Yat-sen Memorial Hospital, Sun Yat-sen University, Guangzhou, China; ^2^Guangzhou Key Laboratory of Molecular Mechanism and Translation in Major Cardiovascular Disease, Guangzhou, China; ^3^Guangdong Province Key Laboratory of Arrhythmia and Electrophysiology, Guangzhou, China; ^4^Department of Pharmacology and Systems Physiology, University of Cincinnati College of Medicine, Cincinnati, OH, United States

**Keywords:** sex difference, COVID-19, stay-at-home orders, healthcare worker, psychological, health behaviors

## Abstract

**Background:** Females with novel coronavirus disease 2019 (COVID-19) state-ordered home isolation were associated with higher anxiety and reduced sleep quality than males. Sex differences in psychobehavioral changes during the COVID-19 stay-at home orders among healthcare workers remained unclear. The purpose of this study was to explore the sex differences in psychological burden and health behaviors among these persons.

**Methods:** This was a cross-sectional study using online data available in the open Interuniversity Consortium for Political and Social Research (OPENICPSR). Healthcare workers including females and males who transitioned to working from home during the COVID-19 stay-at-home orders were included. Sex differences were compared using the chi-square test and Student's *t*-test. We performed logistic and linear regression analyses to determine the association of females with psychological burden and health behaviors.

**Results:** A total of 537 respondents (425 females and 112 males) were enrolled in our study. Sex differences in age (42.1 ± 12.3 years vs. 46.6 ± 15.7 years, *t* = −2.821, *p* = 0.005), occupation (χ^2^ = 41.037, *p* < 0.001), mood change (*n* = 297, 69.9% vs. *n* = 61, 54.5%, χ^2^ = 9.482, *p* = 0.002), bedtime schedule (χ^2^ = 6.254, *p* = 0.044) and news consumption (*n* = 344, 80.9% vs. *n* = 76, 67.9%, χ^2^ = 8.905, *p* = 0.003) were statistically significant. Logistic regression showed that females was negatively associated with better mood status (OR = 0.586, 95% CI 0.153–2.247, *p* = 0.436). In addition, linear regression showed that females were not correlated with total sleep time after adjusting for sio-demographics, mental health outcomes and health behaviors (*B* = 0.038, 95% CI −0.313–0.388, *p* = 0.833).

**Conclusion:** No sex differences in psychological burden and health behaviors of healthcare workers were found during the COVID-19 stay-at-home orders. The COVID-19 state-ordered home isolation may be a potential way to reduce disproportionate effects of COVID-19 pandemic on females and help to minimize sex differences in psychological burden and health behaviors among healthcare workers.

## Introduction

COVID-19, caused by severe acute respiratory coronavirus 2 (SARS-CoV-2), has been a major public health emergency since the end of January 2020 (1). Rapid transmission of the virus tremendously threatened public health and dramatically challenged healthcare systems across the world (2–4), especially in the US (5). Social distancing policies were enacted from the beginning of March 2020, and many people, including some healthcare workers, were forced to stay at home to reduce the spread of the virus (6, 7). Interestingly, females affected by the COVID-19 state-ordered home isolation were proven to be associated with higher anxiety and reduced sleep quality in the general population (8).

Healthcare workers, a unique population who continued working during the COVID-19 state-ordered home isolation, with their frontline peers directly engaged in the clinical management of patients with COVID-19, are at high risk of mental morbidity (9–11) and negative health behaviors (12–14). A cross-sectional study consisting of 1,257 healthcare workers from China showed that females working in hospitals were predisposed to be psychologically stressed, with greater symptoms of anxiety, depression and distress than their male counterparts (15). Similarly, males in France reported lower occurrence rates of symptoms of anxiety and depression working in intensive care units (ICUs) with severe COVID-19 patients (16). Additionally, changes in health behaviors, including sleep problems, work overload, less exercise, increased smoking and drinking, and unhealthy diets, were commonly reported among healthcare workers during the COVID-19 outbreak (7, 14). The total sleep time was significantly shortened in those who continued working on frontlines (7). The rates of smoking and drinking were higher, and both were conversely proven to be protective against anxiety and depression, leading better mental health finally (14).

However, the sex differences in psychological burden and health behaviors due to COVID-19 state-ordered home isolation among healthcare workers who transitioned to working from home remain unclear. Herein, we sought to investigate the sex differences and hypothesized that the COVID-19 state-ordered home isolation could minimize sex differences and help to reduce psychological burden and improve health behaviors of females.

## Methods

### Study Design

**Figure 1:**
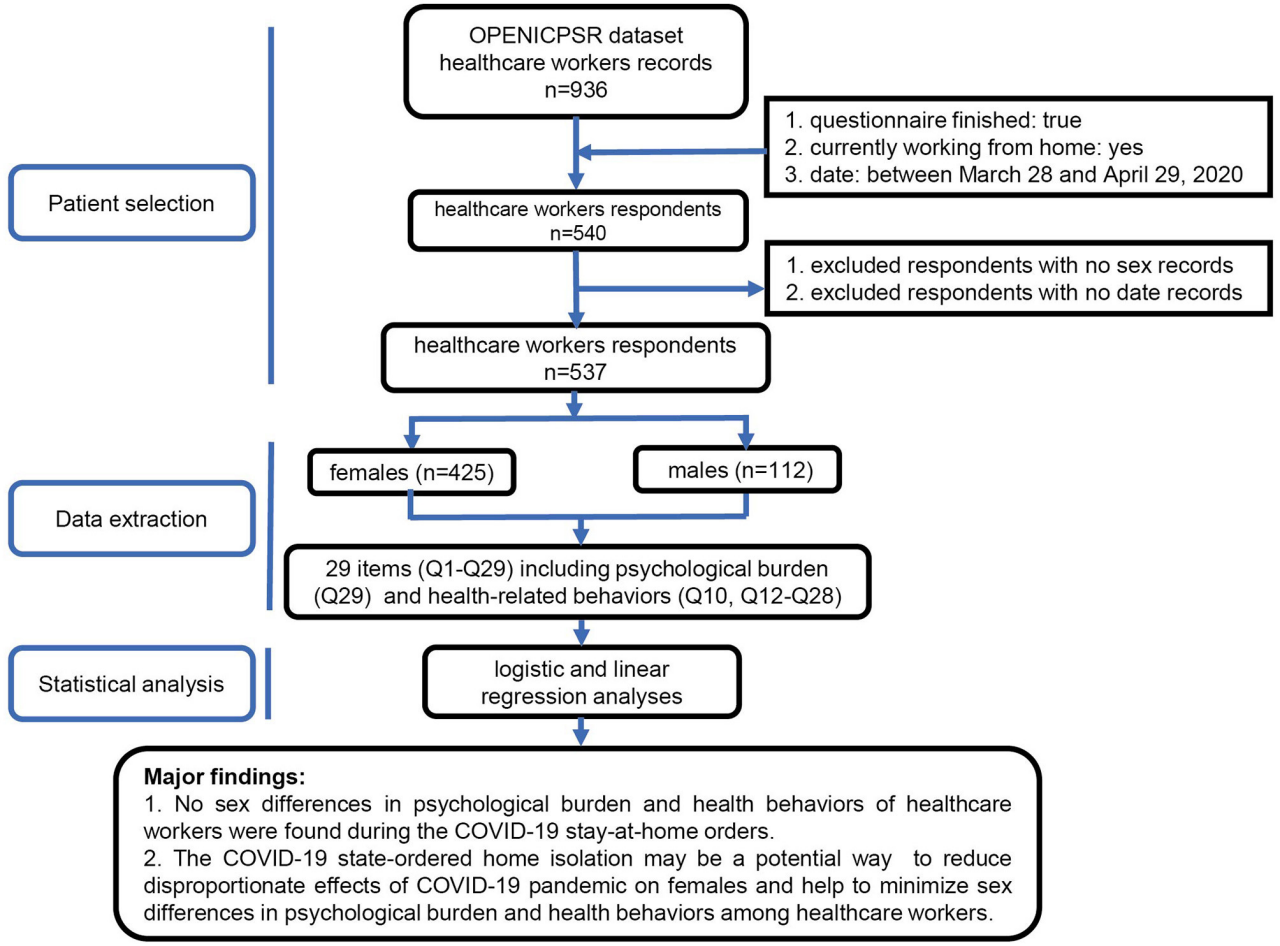
Workflow and major findings of this study. OPENICPSR, open Interuniversity Consortium for Political and Social Research; COVID-19, novel coronavirus disease 2019.

We conducted a cross-sectional, survey-based, region-stratified study using online data. The overall research workflow is depicted in Figure 1. Thereinto, patient selection, data extraction, and statistical analysis were employed. A brief description is as follow. First, we selected the healthcare workers who reported transition of work to home based on the inclusion and exclusion criteria. The records for sex and related items were then extracted from the respondents selected. Next, logistic and linear regression analyses were performed to determine the association of females with psychological burden and health behaviors. Finally, we concluded that there were no sex differences in psychological burden and health behaviors among healthcare workers.

### Data Source

The data was collected by means of online research consisting of 29 items (Q1-Q29) in the questionnaire, and was available in the open Interuniversity Consortium for Political and Social Research (OPENICPSR, https://www.openicpsr.org/openicpsr). Totally, this survey contains nine main items, in which 5 questions are related to health behaviors (Q10, Q12-Q28) including sleep time and schedule, work time and schedule, COVID-19-related media exposure, physical activity and diet; while the others are mainly about the psychological burden (Q29) i.e., mental health outcomes (Q29a, Q29b, Q29c, and Q29d) in the questionnaire. Most questions are yes/no and multiple-choice, except for total sleep time before and after stay-at-home orders, screen time before bed and their occupations. The main question about their mood status was “Please tell us how your mood has changed.” We converted this item as bivariate variables as better/worse, same as before. Detailed information about the study design of the online research have been previously reported (7). This research was approved by the University of Michigan Institutional Review Board (HUM00180147), and studies using the dataset are granted a waiver of informed consent.

### Patient Selection

A total of 936 records about the anonymous responses were collected. And *n* = 834 completed the survey. Then the 834 individuals were included in preliminary analysis by *Conroy DA* et al. The effects of COVID-19 stay-at-home order on sleep, health, and working patterns were compared between healthcare workers who transitioned working from home and those who continued working in person. While in this study, all participants were selected based on the following inclusion criteria: (1) healthcare workers with completed questionnaires, including demographic and psychobehavioral records; (2) healthcare workers currently working from home; and (3) job conducted from home between March 28 and April 29, 2020. Ultimately, 399 individuals were excluded because of no completion of the questionnaire (*n* = 102), no transition to working from home (*n* = 294) and no disclosure of their sex information (*n* = 3), leaving 537 healthcare workers enrolled in this study. It is worth noting that the investigators were not involved in our present study. The mood status records and other variables, including sex, age, occupation, total sleep time and sleep schedule, work time and schedule, media exposure, substance consumption and exercise, were extracted and compared.

### Statistical Analysis

Normally distributed continuous variables including age, total sleep time before and after stay-at-home orders, and screen time before bed are expressed as means ± standard deviations, while other parameters are presented as numbers (percentages). Baseline characteristics were summarized based on their sexes. Both baseline data and sex differences focused on the variables of psychological burden and health behaviors were compared using the chi-square test in this study. We performed logistic and linear regression to determine odds rations (ORs) for the association of females with psychological burden and total sleep time after, respectively. Adjusted variables included age, occupation, mood change, bedtime, news time, ethnicity, race, care for COVID-19 patients directly, total sleep time before, work hours, work schedule, work schedule change, and screen time before bed. Moreover, the mood status (better) was adjusted for total sleep time after and the total sleep time after was adjusted for mood status (better) in turn. All statistical analyses were performed by using SPSS Statistics version 26.0 (IBM) software. A two-sided *P*-value < 0.05 was considered statistically significant.

## Results

### Baseline Characteristics of the Included Healthcare Workers During the COVID-19 Stay-at-Home Orders by Sex

During the first 31 days after implementing the COVID-19 stay-at-home orders in the US, a total of 537 healthcare workers working from home, including 425 (79.1%) females and 112 (20.9%) males, completed the survey. Only 3.4% of the sample reported to care for COVID-19 patients directly. The average and standard deviation of the 537 respondents age was 43 ± 13.2 years, and the majority were non-Latino (n = 511, 95.2%). Of these 537 individuals, 149 (27.7%) were psychologists, 78 (14.5%) were physicians, and 74 (13.8%) were researchers.

Descriptive sio-demographic characteristics of the 537 participants based on sexes are shown in Table 1. Respectively, 2.6% (*n* = 11) of the females and 6.3% (*n* = 7) of the male counterparts were reported to be once engaged in the clinical managements of patients with COVID-19 (χ^2^ = 3.162, *p* = 0.206). The average and standard deviation of females age was 42.1 ± 12.3 years, younger than that of males with 46.6 ± 15.7 years (*t* = −2.821, *p* = 0.005). Among these female healthcare workers who continued working from home, 27.1% were psychologists, 9.9% were physicians and 14.6% were researchers; while 30.4% of the males were psychologists, 32.1% were physicians and 10.7% were researchers (χ^2^ = 41.037, *p* < 0.001). The total sleep time before staying at home was 7.22 ± 0.91 h in females, which was similar to 7.07 ± 0.71 h in males (*t* = 1.835, *p* = 0.068). No significant sex differences were found in ethnicity (χ^2^ = 0.609, *p* = 0.435), race (χ^2^ = 3.200, *p* = 0.202), children at home (χ^2^ = 0.726, *p* = 0.696), alcohol consumption (χ^2^ = 8.922, *p* = 0.112) and date of stay-at-home (χ^2^ = 0.4.461, *p* = 0.347).

**Table 1:** Baseline characteristics of the included healthcare workers during the COVID-19 stay-at-home orders by sex.

**Characteristics**		**Overall**	**Female**	**Male**	***t*** **(*p*-value) or χ^2^ test of independence**
		**Mean ± SD or *n* (%)**	**Mean ± SD or *n* (%)**	**Mean ± SD or *n* (%)**	
Subjects		537 (100.0)	425 (79.1)	112 (20.9)	
Age, years		43.0 ± 13.2	42.1 ± 12.3	46.6 ± 15.7	*t* = −2.821, df = 149, ***p*** **= 0.005**
Ethnicity					χ^2^(1) = 0.609, *p* = 0.435
	Non-Latino	511 (95.2)	406 (95.5)	105 (93.8)	
	Others	26 (4.8)	19 (4.5)	7 (6.3)	
Race					χ^2^(2) = 3.200, *p* = 0.202
	White	456 (84.9)	363 (85.4)	93 (83.0)	
	Asian	34 (6.3)	23 (5.4)	11 (9.8)	
	others	47 (8.8)	39 (9.2)	8 (7.1)	
Occupation					χ^2^(3) = 41.037, ***p*** **< 0.001**
	Psychologist	149 (27.7)	115 (27.1)	34 (30.4)	
	Physician	78 (14.5)	42 (9.9)	36 (32.1)	
	Researcher	74 (13.8)	62 (14.6)	12 (10.7)	
	Others	236 (43.9)	206 (48.5)	30 (26.8)	
Care for COVID-19 patients					χ^2^(2) = 3.162, *p* = 0.206
	Yes	18 (3.4)	11 (2.6)	7 (6.3)	
	No	216 (40.2)	172 (40.5)	44 (39.3)	
	Not provided	303 (56.4)	242 (56.9)	61 (54.5)	
Children at home					χ^2^(2) = 0.726, *p* = 0.696
	Yes	220 (41.0)	175 (41.2)	45 (40.2)	
	No	128 (23.8)	98 (23.1)	30 (26.8)	
	Not applicable	189 (35.2)	152 (35.8)	37 (33.0)	
Alcohol consumption					χ^2^(5) = 8.922, *p* = 0.112
	Never	92 (17.1)	75 (17.6)	17 (15.2)	
	Once a month or less	119 (22.2)	100 (23.5)	19 (17.0)	
	2–4 times a month	163 (30.4)	128 (30.1)	35 (31.3)	
	2–3 times a week	121 (22.5)	93 (21.9)	28 (25.0)	
	4 or more times a week	37 (6.9)	24 (5.6)	13 (11.6)	
Total sleep time, hours					*t* = 1.835, df = 219, *p* = 0.068
	Before stay-at-home	7.19 ± 0.88	7.22 ± 0.91	7.07 ± 0.71	
Date of stay-at-home					χ^2^(4) = 4.461, *p* = 0.347
	March 1–7	29 (5.4)	20 (4.7)	9 (8.0)	
	March 8–14	101 (18.8)	78 (18.4)	23 (20.5)	
	March 15–21	245 (45.6)	201 (47.3)	44 (39.3)	
	March 22–28	106 (19.7)	80 (18.8)	26 (23.2)	
	March 29-April 4	56 (10.4)	46 (10.8)	10 (8.9)	

*Normally distributed continuous variables including age, total sleep time are presented as the mean ± standard deviation and are compared using T-test. Remaining categorical variables were presented as numbers (percentages) and compared using chi-square test. COVID-19, novel coronavirus disease 2019. P < 0.05 was considered significant and presented in bold*.

### Sex Differences in Psychological Burden of Healthcare Workers During the COVID-19 Stay-at-Home Orders

Most healthcare workers changed their mood status during the COVID-19 stay-at-home orders, with higher prevalence of 69.9% (*n* = 297) in females and relatively lower prevalence of 54.5% (*n* = 61) in males (χ^2^ = 9.482, *p* = 0.002). As presented in Table 2, most of them who reported that their mood changed were predisposed to experience worse moods regardless of their sexes (female: *n* = 254, 85.5% vs. male: *n* = 51, 83.6%), while only few of them (female: *n* = 43, 14.5% vs. male: *n* = 10, 16.4%) reported to be better (χ^2^ = 0.147, *p* = 0.701). Of those females whose mood worsened, 24.4% (*n* = 62) had varying degrees of anxiety, 6.7% (*n* = 17) had depression, 7.1% (*n* = 18) were irritable, 27.2% (*n* = 69) had two of them, and 34.6% (*n* = 88) had all of the above. As for the males whose mood worsened, 21.6% (*n* = 11) had varying degrees of anxiety, 5.9% (*n* = 3) had depression, 15.7% (*n* = 8) were irritable, 25.5% (*n* = 13) had two of them, and 31.4% (*n* = 16) had all of the above (χ^2^ = 3.479, *p* = 0.481). Once more, we emphasized that although higher occurrence of mood change was identified among female healthcare workers, there were no particular differences in psychological burden with males.

**Table 2:** Sex differences in psychological burden and health behaviors of healthcare workers during the COVID-19 stay-at-home orders.

**Variables**		**Overall**	**Female**	**Male**	***t or χ^2^*** **(*****p*****-value)**
		**Mean ± SD or *n* (%)**	**Mean ± SD or *n* (%)**	**Mean ± SD or *n* (%)**	
**Psychological burden**					
Mood change					χ^2^(1) = 9.482, ***p*** **= 0.002**
Same as before		179 (33.3)	128 (30.1)	51 (45.5)	
Better/worse		358 (66.7)	297 (69.9)	61 (54.5)	χ^2^(1) = 0.147, *p* = 0.701
Better		53 (14.8)	43 (14.5)	10 (16.4)	χ^2^(1) = 0.135, *p* = 0.713
	Mild-to moderate better	42 (79.2)	35 (81.4)	7 (70.0)	
	Much better	11 (20.8)	8 (18.6)	3 (30.0)	
Worse		305 (85.2)	254 (85.5)	51 (83.6)	χ^2^(4) = 3.479, *p* = 0.481
	Anxious	73 (23.9)	62 (24.4)	11 (21.6)	
	Depressed	20 (4.3)	17 (6.7)	3 (5.9)	
	Irritable	26 (8.5)	18 (7.1)	8 (15.7)	
	Two of them	82 (26.9)	69 (27.2)	13 (25.5)	
	All of the above	104 (34.1)	88 (34.6)	16 (31.4)	
**Health behaviors**					
1. Total sleep time, hours					*t* = 0.240, df = 535, *p* = 0.810
	During stay-at-home	7.22 ± 1.25	7.21 ± 1.33	7.18 ± 1.17	
2. Sleep schedule change					
Bedtime		385 (76.7)	341 (73.9)	71 (63.4)	χ^2^(2) = 6.254, *p* = **0.044**
	Bedtime later	246 (63.9)	199 (63.4)	47 (66.2)	
	Bedtime earlier	40 (10.4)	28 (8.9)	12 (16.9)	
	Bedtime same	99 (25.7)	87 (27.7)	12 (16.9)	
Waketime		386 (71.9)	315 (74.1)	71 (63.4)	χ^2^(2) = 1.797, *p* = 0.407
	Waketime later	285 (73.8)	229 (72.7)	56 (78.9)	
	Waketime earlier	51 (13.2)	45 (14.3)	6 (8.5)	
	Waketime same	50 (13.0)	41 (13.0)	9 (12.7)	
3. Work hours		266 (49.5)	205 (48.2)	61 (54.4)	χ^2^(1) = 1.619, *p* = 0.203
	More hours	69 (25.9)	57 (27.8)	12 (19.7)	
	Fewer hours	197 (74.1)	148 (72.2)	49 (80.3)	
4. Work schedule					χ^2^(1) = 1.919, *p* = 0.166
	Fixed	271 (50.5)	221 (52.0)	50 (44.6)	
	Not fixed	266 (49.5)	204 (48.0)	62 (55.4)	
5. Work schedule change		532 (99.1)	420 (98.8)	112 (100.0)	χ^2^(1) = 1.860, *p* = 0.173
	Yes	312 (58.6)	240 (57.1)	72 (64.3)	
	No	220 (41.4)	180 (42.9)	40 (35.7)	
6. Work-time		228 (42.4)	175 (41.2)	53 (47.3)	χ^2^(1) = 3.211, *p* = 0.073
	Starting work earlier	70 (30.7)	59 (33.7)	11 (20.8)	
	Starting work later	158 (69.3)	116 (66.3)	42 (79.2)	
7. End-time		216 (40.2)	176 (41.4)	40 (35.7)	χ^2^(1) = 0.760, *p* = 0.383
	Ending work earlier	100 (46.3)	79 (44.9)	21 (52.5)	
	Ending work later	116 (53.7)	97 (55.1)	19 (47.5)	
8. News time					χ^2^(1) = 8.905, ***p*** **= 0.003**
	More	420 (78.2)	344 (80.9)	76 (67.9)	
	Less	117 (21.8)	81 (19.1)	36 (32.1)	
9. COVID-19 news time					χ^2^ (4) = 0.613, *p* = 0.962
	0–0.5 h	87 (16.2)	70 (16.5)	17 (15.2)	
	0.5–1 h	167 (31.1)	131 (30.8)	36 (32.1)	
	1–2 h	172 (32.0)	135 (31.8)	37 (33.0)	
	2–3 h	80 (14.9)	63 (14.8)	17 (15.2)	
	3+ h	31 (5.8)	26 (6.1)	5 (4.5)	
10. Screen time before bed, hours					
	During stay-at-home	1.34 ± 0.88	1.31 ± 0.87	1.42 ± 0.90	*t* = −0.793, df = 314, *p* = 0.428
11. Substance consumption					
Food change		279 (52.0)	233 (54.8)	46 (41.1)	χ^2^(1) = 0.308, *p* = 0.579
	More food	76 (27.2)	65 (27.9)	11 (23.9)	
	Less food	203 (72.8)	168 (72.1)	35 (76.1)	
Food change after		304 (56.5)	252 (59.3)	52 (46.4)	χ^2^(1) = 0.098, *p* = 0.754
	More healthy	146 (48.0)	120 (47.6)	26 (50.0)	
	Less healthy	158 (52.0)	132 (52.4)	26 (50.0)	
Alcohol change					χ^2^(1) = 0.321, *p* = 0.571
	No	362 (67.4)	289 (68.0)	73 (65.2)	
	Yes	175 (32.6)	136 (32.0)	39 (34.8)	
Alcohol change after		175 (32.6)	136 (32.0)	39 (34.8)	χ^**2**^(1) = 1.870, *p* = 0.171
	More	147 (84.0)	117 (86.0)	30 (76.9)	
	Less	28 (16.0)	19 (14.0)	9 (23.1)	
12. Exercise/movement		435 (81.0)	345 (81.2)	90 (80.3)	χ^**2**^(1) = 0.749, *p* = 0.387
	Less	224 (41.7)	174 (50.4)	50 (55.6)	
	More	211 (39.3)	171 (49.6)	40 (44.4)	

*Normally distributed continuous variables including total sleep time, and screen time before bed are presented as the mean ± standard deviation and are compared using T-test. Remaining categorical variables were presented as numbers (percentages) and compared using chi-square test. COVID-19, novel coronavirus disease 2019. P < 0.05 was considered significant and presented in bold*.

### Sex Differences in Health Behaviors of Healthcare Workers During the COVID-19 Stay-at-Home Orders

#### Sex Differences in Total Sleep Time and Schedule

Sex differences in health behaviors, including sleep time and schedule, work patterns, media exposure and screen time before bed, food and alcohol consumption, and exercise frequency, were assessed in Table 2. Differences in bedtime (χ^2^ = 6.254, *p* = 0.044) between sexes was significantly noted. The females and males estimated their bedtime to be later (*n* = 199, 63.4% vs. *n* = 47, 66.2%), earlier (*n* = 28, 8.9% vs. *n* = 12, 16.9%), or the same as before (*n* = 87, 27.7% vs. *n* = 12, 16.9%). However, the total sleep times for females and males was not significantly different (7.21 ± 1.33 h vs. 7.18 ± 1.17 h, *t* = 0.240, *p* = 0.810).

#### Sex Differences in Work Patterns

Table 2 shows that 221 (52.0%) females were required to follow a fixed work schedule when working from home, while over half (*n* = 62, 55.4%) of the males had a non-fixed schedule (χ^2^ = 1.919, *p* = 0.166). As a result, 64.3% of the males adjusted their working schedules (χ^2^ = 1.860, *p* = 0.173). A total of 79.2% of them started work later (χ^2^ = 3.211, *p* = 0.073), and 52.5% ended work earlier (χ^2^ = 0.760, *p* = 0.383), which was not different from females. Eliminating the missing data, 72.2% (*n* = 148) of the females and 80.3% (*n* = 49) of the males similarly worked for fewer hours (χ^2^ = 1.619, *p* = 0.203).

#### Sex Differences in Media Exposure and Screen Time Before Bed

Despite of the fact that females consumed more news time than males (*n* = 344, 80.9% vs. *n* = 76, 67.9%, χ^2^ = 8.905, *p* = 0.003), the average and standard deviation of media time regarding COVID-19 was not significantly different. Majorities of them in both groups (*n* = 266, 62.6%, and *n* = 73, 65.1%, respectively) tended to consume 0.5–2 h each day (χ^2^ = 0.613, *p* = 0.962). The average screen time before bed in females was 1.31 ± 0.87 h, which was similar to 1.42 ± 0.90 h in males (*t* = −0.793, *p* = 0.428).

#### Sex Differences in Substance Consumption and Exercise

Finally, there were no sex differences in food consumption (χ^2^ = 0.308, *p* = 0.579), food quality (χ^2^ = 0.098, *p* = 0.754), and alcohol consumption (χ^2^ = 1.870, *p* = 0.171) during the COVID-19 stay-at-home orders. Furthermore, over half of the healthcare workers (*n* = 174, 50.4% of females and *n* = 50, 55.6% of males) exercised less when staying at home, with no sex difference (χ^2^ = 0.749, *p* = 0.387).

### Logistic and Linear Analyses Determining the Association of Females With Mood Status and Total Sleep Time During the COVID-19 Stay-at-Home Orders

Logistic and linear regression analyses were performed in Table 3 to determine the association of females with mood status and total sleep time during the COVID-19 stay-at-home orders. Findings showed that in logistic regression analysis, females had no relationship with better mood status (OR = 1.148, 95% CI 0.558–2.363, *p* = 0.708), even after adjusting for age and occupation in model 1 (OR = 1.002, 95% CI 0.980–1.024, *p* = 0.857), with further adjustment for mood change, bedtime, and news time in model 2 (OR = 0.962, 95% CI 0.413–2.239, *p* = 0.928), with further adjustment for ethnicity, race, care for COVID-19 patients, total sleep time before, work hours, work schedule, work schedule change and screen time before bed in model 3 (OR = 0.760, 95% CI 0.204–2.828, *p* = 0.682), and finally with further adjustment for total sleep time after in model 4 (OR = 0.586, 95% CI 0.153–2.247, *p* = 0.436). In addition, in linear regression analysis, females were not correlated with total sleep time after adjustments for age, occupation, mood change, bedtime, news time, ethnicity, race, care for COVID-19 patients, total sleep time before, work hours, work schedule, work schedule change, screen time before bed and mood status (*B* = 0.038, 95% CI −0.313–0.388, *p* = 0.833).

**Table 3:** Logistic and linear analyses determining the association of females with mood status and total sleep time during the COVID-19 stay-at-home orders.

**Variable**	**Mood status (better)**		**Total sleep time after**	
	**OR (95% CI)**	***P*** **-value**	**B (95% CI)**	***P*** **-value**
Unadjusted	1.148 (0.558–2.363)	0.708	0.033 (−0.238–0.304)	0.810
Model 1#	1.002 (0.980–1.024)	0.857	0.048 (−0.225–0.321)	0.729
Model 2*	0.962 (0.413–2.239)	0.928	0.104 (−0.171–0.379)	0.459
Model 3^@^	0.760 (0.204–2.828)	0.682	0.033 (−0.323–0.389)	0.854
Model 4^∧^	0.586 (0.153–2.247)	0.436	0.038 (−0.313–0.388)	0.833

# *Adjusted for age, occupation*.

* *Adjusted for age, occupation, mood change, bedtime, news time*.

@ *Adjusted for age, occupation, mood change, bedtime, news time, ethnicity, race, care for COVID-19 patients, total sleep time before, work hours, work schedule, work schedule change, screen time before bed*.

∧ *Adjusted as model 3 with further adjustment for total sleep time after in logistic regression analysis and with further adjustment for mood status in linear regression analysis*.

*Logistic regression was performed to determine the association between females and mood status. Linear regression was used to illustrate the association between females and total sleep time after. OR, Odds ratio; COVID-19, novel coronavirus disease 2019. P < 0.05 was considered significant*.

## Discussion

This is the first study to illustrate sex differences in mental and physical impacts of the COVID-19 state-ordered home isolation on healthcare workers. The major findings are summarized as follows: (1) No sex differences in psychological burden and health behaviors of healthcare workers were found during the COVID-19 stay-at-home orders. (2) The COVID-19 state-ordered home isolation may be a potential way to reduce disproportionate effects of COVID-19 pandemic on females and help to minimize sex differences in psychological burden and health behaviors among healthcare workers.

The psychological and behavioral responses among healthcare workers in this study were consistent with previous studies (17, 18), but sex-stratified differences were not quite the same as those in the general population during the COVID-19 stay-at-home orders (8). Connor et al. has reviewed multi-factors including health, economic and social systems that could contribute to exacerbated sex differences in health risks and outcomes on females, and implicated that such differences could be expanded during the COVID-19 pandemic (19). Female healthcare workers, serving as the mainstream of healthcare workforce who were at high risk of SARS-CoV-2 exposure has been proven to be disproportionately affected by the shortage of personal protective equipment, limited testing capacity and increased unemployment during the COVID-19 outbreak (2, 16, 20, 21). Thus, the COVID-19 stay-at-home orders was implemented aiming to reduce the risks posed by COVID-19 pandemic. And the online research initiated by Conroy et al. have demonstrated that both mood status and total sleep time were virtually improved in overall population regardless of their sexes during the COVID-19 stay-at-home orders, but were unfortunately identified to be associated with higher anxiety and reduced sleep quality in female population (8). However, recent studies focused on sex differences in the US and worldwide revealed controversial opinions. For example, some studies suggested a high prevalence of psychological symptoms in females (15, 16), some indicated a higher prevalence in males (22, 23), while others showed no difference, as in this study (24, 25). A parallel study consisting of 103 participants launched in the US identified a stronger association between females and stay-at-home anxiety (8). Among the participants, there were 61 (59.2%) females and 42 (40.8%) males, with a lower percentage of females compared with our study. The average age of the females was less than 40 years and they potentially possessed less working experience. Previous studies have suggested that age is a critical determinant of mental morbidity. In detail, young adults aged 18–49 years are more likely to develop anxiety than older adults aged >50 years (26). Although the females in our study were younger than the males, both were older than 40 years, indicating that more working experience matters when faced with such an unprecedented time like the COVID-19 pandemic. Besides, more females were reported to be unemployed (62.2 vs. 37.8%) and laid off (62.5 vs. 37.5%) than males in this parallel study, making themselves struggling with severe economic stress. While in our research, 85.4% of the females were white, and 95.5% were non-Latino. They represented middle-to-high levels of income and were less likely to experience inadequate health insurance, financial stress, and caregiving burden (19) and thus were less likely to experience psychological burden. Moreover, females serving as medical and domestic caregivers were proven to experience a higher prevalence of social isolation and spiritual distress during home isolation (27) and were predisposed to develop symptoms of anxiety in the early phase of the pandemic and depression in the repair phase (10, 28, 29). Xiao et al. have verified that social support was capable to influence anxiety during home isolation. And the anxiety could further act as a medicator between social isolation and sleep disturbance (30). It's noting that this cross-sectional study included 180 healthcare workers who treated patients with COVID-19 in January and February 2020 in Wuhan, China. While in our study, only 3.4% of the sample were engaged in caring for COVID-19 patients directly, which means reduced risks posed by COVID-19 outbreak and helps to elucidate negative results of disproportionate effects on females in our study.

Likewise, a web-based cross-sectional survey incorporating 7,236 respondents including 3,952 (54.6%) females and 3,284 (45.4%) males implied that there were no sex differences in depressive symptoms and sleep quality during the COVID-19 outbreak (25). Liu et al. also showed negative association of females with symptoms of depression and anxiety among young adult individuals aging 18–30 years in the US (24). More interestingly, another survey study engaging 1,210 respondents including 3,437 (60.3%) males reported higher prevalence of stress and anxiety in males (22). These included respondents were patients hospitalized with confirmed COVID-19. And this population were thought to have more risks posed by the COVID-19 outbreak to develop disproportionate effects on females. However, the results were dramatically opposite. More researches are needed to identify whether the females are susceptible to be disproportionately affected during the unprecedent time. In the present study, we found that more females reported mood change during the COVID-19 stay-at-home orders, which may be helpful to eliminate such disproportionate effects posed by the COVID-19 pandemic. Further studies are necessary to determine the association of females with psychological burden after implementing the COVID-19 state-ordered home isolation.

In addition, changes of total sleep time, sleep quality and sleep schedules were essential parts of health behaviors during the COVID-19 stay-at-home orders. Contrary to our hypothesis, we did not observe sex difference in total sleep time after enacting the COVID-19 state-ordered home isolation. While some studies showed positive findings (7, 31), some hold opposite views (8, 9), while others identified no sex differences as presented in our study (30). Compared with healthcare workers who continued working on the frontlines, Conroy et al. found that the total sleep time was longer in those who transitioned to working from home during the COVID-19 state-ordered home isolation (7). Similar findings were found in the population of university students that the total sleep time increased significantly in weekdays and weekends during the COVID-19 stay-at-home orders than before (31). However, the sleep quality was not different between sexes in healthcare workers and in general population (8, 31), which is consistent with our findings in this research. It's noting that we identified significant difference in bedtime schedule between females and males, but there are still no particular association of females with total sleep time after adjustment. Besides, alcohol consumption was reported to be associated with better mental health in healthcare workers because it helps to relieve mental stress (14). But it is quite disputed for smoking. Previous studies have illustrated that cigarettes may help to relieve negative emotions such as anxiety and stress (32). A cross-sectional study of 7,124 healthcare workers in 19 hospitals and health centers in Vietnam has confirmed that smoking was related to lower anxiety and depression likelihood during the COVID-19 pandemic (14). While other studies found that daily smoking contributed to extending influence on mental stress (33). Currently, smoking and its influence on females are still controversial (34). In the sex-stratified analysis, the association of perceived stress with smoking and alcohol consumption was similar between females and males (32, 35), which is concordant with our findings. Furthermore, we observed sex differences in occupation status and news time before bed. All findings indicated no specific association of females with mood status and total sleep time after adjustments. Finally, we also present concerns about the workplace environment and its impact on healthcare workers during the COVID-19 stay-at-home orders (36). Medical staff in hospital workplace conditions are susceptible to develop fatigue, which is associated with increased anxiety and emotional stress (37). Previous studies have demonstrated that such impacts can be mitigated during the COVID-19 stay-at-home orders (7). However, the sex difference in indirect (mediating) effects of non-hospital workplace conditions were not the primary question in this study. Further investigation is needed to determine the association with workplace culture, a potential mediator in sex differences of psychological burden and health behaviors.

### Limitations

There were some limitations in our study. First, it was confined in terms of ethnic scope. Healthcare workers were mostly non-Latino whites living in Michigan, and thus, it was limited to reflecting the interactive effects between sex and ethnicity (particularly Black, Latinx, low-income, and immigrant populations). Second, this research was simply focused on immediate changes of psychobehavioral responses from March 28 to April 29, 2020, ~4 weeks after the implementation of the COVID-19 stay-at-home orders. Therefore, sex-stratified long-term differences in mental and physical implications among this population are worth further investigation.

### Perspectives and Significance

Contrary to previous findings, there are insufficient evidence supporting sex differences in psychological burden and health behaviors during the COVID-19 stay-at-home orders. The disproportionate effects of COVID-19 pandemic on females no longer existed, indicating that the distancing intervention i.e., the COVID-19 state-ordered home isolation may be a potential way to minimize sex differences among healthcare workers. Eliminating sex differences is an important step to maintain healthcare workforce during such unprecedented times. More policies, like the COVID-19 state-ordered home isolation, are needed to promote the recovery of the mentally and physically documented posttraumatic effects on females.

## Data Availability Statement

Publicly available datasets were analyzed in this study. This data can be found here: https://www.openicpsr.org/openicpsr.

## Ethics Statement

The dataset was approved for research use by the University of Michigan Institutional Review Board (HUM00180147) and studies using the dataset are granted a waiver of informed consent. All methods were performed in accordance with the relevant guidelines and regulations.

## Author Contributions

YZ and JW contributed to conception and design of the study. WG, XL, and RS had full access to all data in the study and take responsibility for the integrity of the data and accuracy of the data analysis. XL and WG performed the statistical analysis. WG and RS wrote the first draft of the manuscript. YJ, ZCa, MW, JM, and ZCh wrote sections of the manuscript. All authors contributed to manuscript revision, read, and approved the submitted version.

## Funding

This work was supported by the National Natural Science Foundation of China (JW, 82070237; YZ, 81870170; YC, 81970200; YC, 81770229; RS, 81700397; and XL, 82100347), Natural Science Foundation of Guangdong Province (YZ, 2019A1515011682), National High Technology Research and Development Program of Guangzhou (JW, 20180304001; JW, 2019GZR110406004; and YZ, 201704020044), China Postdoctoral Science Foundation (ZCa, 2020M683123), Guangdong Basic and Applied Basic Research Foundation (MW, 2019A1515110129), and Guangdong Medical Science and Technology Research Foundation (YJ, A2021006). All of the fundings had no role in design, methods, subject recruitment, data collections, analysis, and preparation of paper. We acknowledge the grant support from Guangzhou Science Technology Bureau (202102010007).

## Conflict of Interest

The authors declare that the research was conducted in the absence of any commercial or financial relationships that could be construed as a potential conflict of interest.

## Publisher's Note

All claims expressed in this article are solely those of the authors and do not necessarily represent those of their affiliated organizations, or those of the publisher, the editors and the reviewers. Any product that may be evaluated in this article, or claim that may be made by its manufacturer, is not guaranteed or endorsed by the publisher.

## Abbreviations

COVID-19: novel coronavirus disease 2019
the US: the United States
ICU: intensive care unit
SARS: severe acute respiratory syndrome.
